# The impact of recurrent mitral regurgitation after surgical or transcatheter mitral valve repair: a comprehensive review and a meta-analysis

**DOI:** 10.1093/icvts/ivaf109

**Published:** 2025-04-29

**Authors:** Chengyuan Yu, Arian Arjomandi Rad, Hongbo He, Yichen Yang, Jos G Maessen, Peyman Sardari Nia

**Affiliations:** Department of Cardiothoracic Surgery, Maastricht University Medical Centre, Maastricht, The Netherlands; Department of Cardiothoracic Surgery, Maastricht University Medical Centre, Maastricht, The Netherlands; Medical Sciences Division, University of Oxford, Oxford, UK; Department of Cardiothoracic Surgery, Maastricht University Medical Centre, Maastricht, The Netherlands; Department of Cardiothoracic Surgery, Maastricht University Medical Centre, Maastricht, The Netherlands; Department of Cardiothoracic Surgery, Maastricht University Medical Centre, Maastricht, The Netherlands; Department of Cardiothoracic Surgery, Maastricht University Medical Centre, Maastricht, The Netherlands

**Keywords:** recurrent mitral regurgitation, primary mitral regurgitation, secondary mitral regurgitation, mitral valve repair, transcatheter mitral valve repair, meta-analysis

## Abstract

**BACKGROUND:**

Recurrent mitral regurgitation (MR) is typically defined as MR that is moderate or severe on follow-up echocardiography after the intervention. This meta-analysis summarizes the results of all available studies on the impact of recurrent MR on clinical outcomes after the intervention.

**METHODS:**

Medline, EMBASE, PubMed and Web of Science were searched from January 2000 to August 2024 for original studies reporting outcomes about the clinical impact of recurrent MR. Five clinical outcomes were analysed: reoperation, cardiovascular deaths, readmission, heart failure and New York Heart Association (NYHA) functional classification. The summary odds ratio (OR) with the 95% confidence interval (CI) was used to assess the risk of clinical outcomes.

**RESULTS:**

A total of 22 studies were included in the final analysis, involving 5,804 patients, of which 960 had recurrent MR. The overall pooled incidence of recurrent MR is 16.54%. Secondary or primary patients with MR with recurrent MR after an intervention had higher rates of reoperation [(OR = 6.25, 95% CI, 2.95–14.41; *P* < 0.001) or (OR = 22.54, 95% CI, 14.96–33.98; *P* < 0.001)]or of cardiovascular death [(OR = 5.26, 95% CI, 2.35–11.77; *P* < 0.001) or (OR = 1.68, 95% CI, 1.32–2.14; *P* < 0.001)]. The rates were also high for readmission (OR = 3.95, 95% CI, 2.56–6.10; *P* < 0.001), heart failure incidence (OR = 2.87, 95% CI, 1.75–5.11; *P* < 0.001) and the number of NYHA functional class III/IV (OR = 5.40, 95% CI, 3.01–9.70; *P* < 0.001) for recurrent MR of secondary MR. However, no significant association was found between recurrent MR of primary MR and the incidence of NYHA functional class III/IV (OR = 1.02, 95% CI, 0.47–2.22; *P* = 0.96).

**CONCLUSIONS:**

Recurrent MR is associated with higher rates of reoperations, readmissions, cardiovascular deaths, incidences of heart failure and NYHA functional class III/IV numbers. However, recurrent MR of primary MR is not correlated with NYHA functional class III/IV.

## GRAPHICAL ABSTRACT

**Figure:**
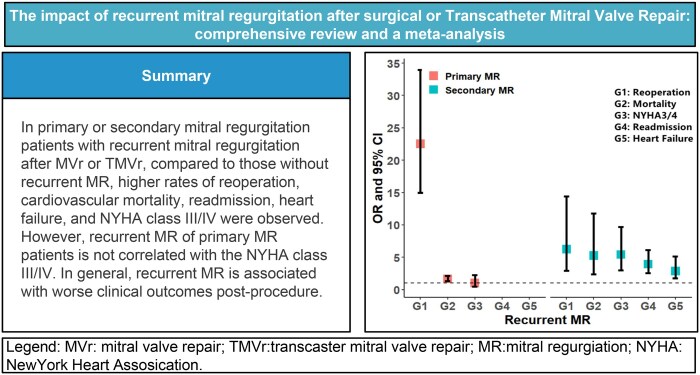

## INTRODUCTION

Mitral regurgitation (MR) is one of the most common types of valvular heart disease [1]. It is classified into 2 categories based on the underlying mechanisms: primary MR, which is an intrinsic disease of the valve apparatus, like mitral valve prolapse, and secondary MR caused by extrinsic processes that affect valve function, such as remodelling and dysfunction of the ventricles and the annulus [2–4]. Mitral valve repair (MVr) and transcatheter mitral valve repair (TMVr) are established techniques for treating MR [5, 6]. Compared with mitral valve replacement, MVr is associated with superior long-term survival, lower perioperative morbidity and mortality and also a reduced risk of thromboembolic events [4, 7]. However, the recurrence of MR after mitral valve repair is higher than that after mitral valve replacement, and it might not result in a permanent correction of MR, which could substantially affect long-term outcomes [8–12]. Many reports show that the major limitation of MVr is the lack of durability with recurrent MR [9, 13–15].

Recurrent MR is typically defined as MR that was moderate or severe on follow-up echocardiography after the procedure or discharge from the hospital. In the 2021 European Society of Cardiology/European Association for Cardio-Thoracic Surgery guidelines, moderate-to-severe MR occurring beyond 6 months postoperatively is defined as late recurrent MR, whereas MR within 6 months is classified as early recurrent MR [16]. Given that postoperative MR requires a period of observation to assess its progression and to avoid excessive intervention for transient postoperative regurgitation, clinical practice tends to adopt the 6-month postoperative mark as the formal diagnostic threshold for recurrent MR [17, 18]. The rate of recurrent moderate or severe ischaemic MR after MV repair can reach from about 10% to 30% [18–21]. For patients who have undergone MVr, even mild recurrent MR can pose a risk, and the risk increases with time [11, 18, 22]. In a prior study, MR worsened from grade 1+ or 2+ to grade 3+ or 4+ in 1 year for 11.4% of 220 patients with MVr [21]. Recurrent MR is found to be related to the absence of left ventricle reverse remodelling and unfavourable clinical outcomes in several observational studies [4, 23, 24]. Therefore, the goal of this meta-analysis is to provide a comprehensive review and meta-analysis of the impact on clinical outcomes of recurrent MR after intervention (MVr or TMVr).

## MATERIALS AND METHODS

### Protocol and registration

The protocol of this meta-analysis was registered in the International Prospective Register of Systematic Reviews (PROSPERO CRD42024595898 2024). This meta-analysis was conducted according to the PRISMA 2020 guidelines [25]. Ethical approval was not necessary for this work because this was an analysis of previously published data.

### Search strategy

Relevant articles published from January 2000 to August 2024 were retrieved from 4 electronic databases (Medline, EMBASE, PubMed and Web of Science). The search strategy used subject terms and free-text terms combined with Boolean operators (see Supplementary Materials for details). The search terms used were as follows: (recurrent mitral regurgitation) AND (mitral valve repair OR Mitral Valve Annulus Repair OR Mitral Annuloplasties OR Mitral Valve Annuloplasties OR Valve Annuloplasties, Mitral OR Valve Annuloplasty, Mitral OR Mitral Annuloplasty OR transcatheter mitral valve repair). The reference lists of retrieved articles were also screened for potentially relevant articles. The literature search was independently conducted by 2 researchers (C.Y. and H.H.), with any discrepancies resolved through discussion with a third researcher (P.S.).

### Inclusion and exclusion criteria

Articles that directly discuss and research the impact of recurrent MR on patients after MVr during the follow-up period are included. Articles were excluded only if (i) the cumulative incidence of recurrent MR was discussed; (ii) the follow-up of postoperative patients occurred less than 6 months after discharge; (iii) the number of postoperative patients with recurrent MR was less than 10 and (iv) the study included only postoperative patients with less than moderate recurrent MR. Case reports, abstracts only, reviews, care reports or commentaries, duplicates and other irrelevant studies were excluded. Two authors (C.Y. and Y.Y.) screened and assessed the studies independently for inclusion. Disagreements regarding the inclusion of articles were resolved via a discussion with a third researcher (P.S.).

### Data extraction

The titles and abstracts of the retrieved records were initially screened. Studies that did not meet the inclusion criteria were excluded. Full-text versions of all potentially eligible abstracts were retrieved and cross-checked by 2 authors (C.Y. and H.H.) to confirm inclusion. The senior author (P.S.) independently assessed any discrepancies in inclusion decisions to ensure consistency. Data from the selected studies were tabulated, extracted and cross-checked by 2 authors (Y.Y. and H.H.) using a standardized form. This article selected the following data from the included articles: first author, publication year, study design, patient number, follow-up, inclusion period, cardiovascular mortality, heart failure rate, reoperation rate, readmission rate and New York Heart Association (NYHA) cardiac function. Supplementary Table S1 shows the definitions of recurrent MR and outcome standards in each included study.

### Quality assessment

The Newcastle–Ottawa scale, which includes 8 evaluation items, was used to assess the quality of the included studies. The scale includes scores from 0 to 9. Newcastle–Ottawa scale studies with a score of 7 or higher are considered high quality and low risk. This work was independently cross-checked with the original publications for accuracy and completeness by 2 other researchers (H.H and Y.Y.).

### Statistical methods

Stata 17 (StataCorp, College Station, TX, USA) and Review Manager 5.3 (https://revman.cochrane.org/info) were used for data analysis and statistical computations. Utilizing the *I*^2^ value and Cochran’s *Q* statistic to evaluate statistical heterogeneity, an *I*^2^ value of <25%, 25% to 50% or >50% indicates low, moderate or high heterogeneity, respectively. Subgroup analysis was utilized to evaluate heterogeneity in cases with high levels of heterogeneity, and meta-regression analysis was used to investigate the factors that influenced heterogeneity. If significant heterogeneity was observed (*I*^2^ > 50%), pooled estimates were calculated using a random-effects model. Otherwise, a fixed-effect model was used. The funnel plot was visually examined, and the Egger test was used to assess publication bias and small-study effects. The leave-one-out method was applied to perform sensitivity analysis to evaluate the influence of individual studies on the overall estimate. *P*-values < 0.05 were regarded as statistically significant.

## RESULTS

**Figure 1:**
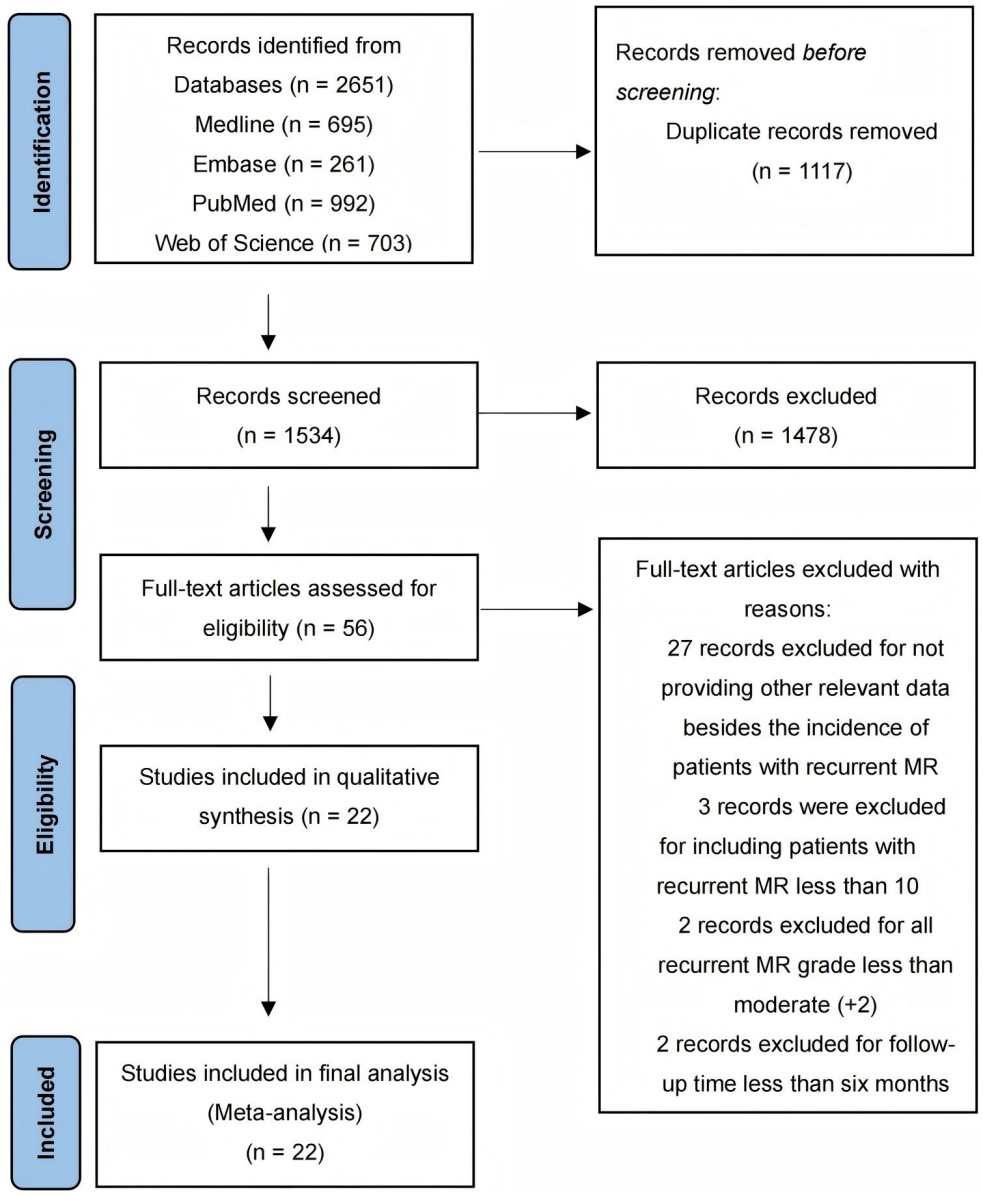
Flow diagram of the studies included in the data search.

A total of 56 studies were found for review through the literature search. Of these, 28 studies were excluded for not providing relevant data other than the incidence of patients with recurrent MR. Three studies were excluded for including fewer than 10 patients with recurrent MR; 2 studies were excluded for all recurrent MR grades less than moderate (+2); 2 studies were excluded for follow-up time less than 6 months. Finally, a total of 22 observational studies [15, 18, 21, 26–44] were found that satisfied the inclusion criteria for this meta-analysis (Fig. 1).

Using the Newcastle-Ottawa Quality Assessment Scale, all non-randomized studies were of good quality (Table 1). In Table 2, we presented the characteristics of these studies, including the first author, publication year, follow-up, number of patients, study design and inclusion period. In 22 studies, 14 were about primary MR and 8 were about secondary MR. There were a total of 5,804 patients, including 960 patients with recurrent MR and 4,844 patients without recurrent MR. In these studies, there were 4,858 patients with primary MR and 946 patients with secondary MR. The overall pooled incidence of recurrent MR is 16.54%, with the pooled incidence for primary MR and secondary MR being 15.39% and 22.41%, respectively. The follow-up periods were approximately 6 months in 1 study [34], around 1 year in 4 studies [21, 28, 30, 37], around 3 years in 3 studies [27, 32, 36], around 5 years in 5 studies [15, 26, 31, 35, 41], around 10 years in 3 studies [29, 33, 39] and more than 10 years in 6 studies [18, 38, 40, 42–44]. Among these patients, 5,392 patients had undergone MVr, 442 had undergone TMVr and 960 experienced recurrent MR≥ 2+ during follow-up. The main characteristics of patients during the follow-up period in the included studies are listed in Table 2. Among the 22 studies, 17 reported reoperations [15, 21, 26–30, 32, 33, 35–39, 41–44]; 12 reported cardiovascular deaths [18, 26, 29, 30, 32, 34–37, 39, 41, 44]; 4 reported readmission rates [15, 21, 26, 28]; 4 reported heart failure [15, 21, 28, 30] and 5 reported NYHA classifications [21, 31, 34, 40, 44]. In 1 report, deaths occurred in patients without recurrent MR, and the results of 6 other reports show that all patients who underwent reoperations had recurrent MR.

**Table 1:** Quality assessment of the nonrandomized studies using the Newcastle-Ottawa scale

Study	Selection				Comparability (based on design and analysis)	Outcome			
	Representativeness of the exposed cohort	Selection of the nonexposed cohort	Ascertainment of exposure	Outcome of interest absent at start of study		Assessment of outcome	Follow-up long enough for outcomes to occur	Adequacy of follow-up of cohorts	Total score
Petrus *et al.*	1	1	1	1	2	1	1	1	9
Suri *et al.*	1	1	1	1	2	1	1	1	9
David *et al.*	1	1	1	1	2	1	1	1	9
Hellhammer *et al.*	1	1	1	1	1	1	1	1	8
Kim *et al.*	1	1	1	1	1	1	1	1	8
Salsano *et al.*	1	1	1	1	1	1	1	1	8
Kaneyuki *et al.*	1	1	1	1	1	1	1	1	8
Zhong *et al.*	1	1	1	1	2	1	1	1	9
Petrus *et al.*	1	1	1	1	1	1	1	1	8
Onorati *et al.*	1	1	1	1	2	1	1	1	9
Lee *et al.*	1	1	1	1	2	1	1	1	9
Magne *et al.*	1	1	1	1	1	1	1	1	8
Hu *et al.*	1	1	1	1	0	1	1	1	7
De Bonis *et al.*	1	1	1	1	1	1	1	1	8
Tomsic *et al.*	1	1	1	1	2	1	1	1	9
De Bonis *et al.*	1	1	1	1	1	1	1	1	7
Taramasso *et al.*	1	1	1	1	1	1	1	1	8
De Bonis *et al.*	1	1	1	1	1	1	1	1	7
David *et al.*	1	1	1	1	2	1	1	1	9
Waikittipong	1	1	1	1	1	1	1	1	8
Trumello *et al.*	1	1	1	1	1	1	1	1	8
Llorens *et al.*	1	1	1	1	0	1	1	1	7

**Table 2:** Characteristics and numbers of studies and patients during the follow-up period in the included studies

Study	Publication year	Follow-up (years)	Design	Inclusion period	Type of surgery	Type of MR
Petrus *et al.* [15]	2019	Median 6.8	Observational, retrospective	2000–2014	MVr	Secondary
Suri *et al.* [18]	2016	Median 11.5	Observational, retrospective	1990–2000	MVr	Primary
David *et al.* [44]	2013	Median 10.4	Observational, prospective	1985–2004	MVr	Primary
Hellhammer *et al.* [21]	2021	1	Observational, retrospective	2010–2019	TMVr	Secondary
Kim *et al.* [29]	2018	8.71 ± 5.58	Observational, retrospective	1990–2015	MVr	Primary
Salsano *et al.* [31]	2023	5	Observational, retrospective	2007–2010	MVr	Secondary
Kaneyuki *et al.* [36]	2019	2.7 ± 2.1	Observational, retrospective	2011–2018	MVr	Primary
Zhong *et al.* [32]	2024	Median 3.28	Observational, retrospective	2010–2022	MVr	Primary
Petrus *et al.* [26]	2018	4.75 ± 3.25	Observational, retrospective	2003–2014	MVr	Secondary
Onorati *et al.* [30]	2009	Mean 1.5 ± 0.14 (range 0.08–4.58)	Observational, retrospective	2004–2008	MVr	Secondary
Lee *et al.* [34]	2009	0.5	Observational, retrospective	NA	MVr	Secondary
Magne *et al.* [28]	2009	Mean 1.5 ± 0.6	Observational, retrospective	NA	MVr	Secondary
Hu *et al.* [27]	2024	2	Observational, retrospective	2018–2021	TMVr	Primary
De Bonis *et al.* [33]	2014	Mean 9.2 ± 4.21	Observational, retrospective	1993–2002	TMVr	Primary
Tomsic *et al.* [35]	2019	Median 6.4	Observational, retrospective	2015–2022	MVr	Primary
De Bonis *et al.* [43]	2014	Mean 11.5 ± 3.73	Observational, retrospective	1991–2004	MVr	Primary
Taramasso *et al.* [37]	2014	Median 1.08 (0.58–2.08)	Observational, retrospective	2008–2013	TMVr	Secondary
De Bonis *et al.* [42]	2012	Mean 10.7 ± 3.1	Observational, retrospective	1993–2000	MVr	Primary
David *et al.* [38]	2005	12	Observational, retrospective	1981–2001	MVr	Primary
Waikittipong [39]	2021	Mean 7.5 ± 4.6	Observational, retrospective	2003–2019	MVr	Primary
Trumello *et al.* [40]	2021	Mean 11.6 ± 5.16	Observational, retrospective	1999–2009	MVr	Primary
Llorens *et al.* [41]	2019	Median 6.4	Observational, retrospective	2015–2022	MVr	Primary

Number of patients		Reoperation		Cardiovascular deaths		Readmission		Heart failure		NYHA III/IV	
NRMR	RMR	NRMR	RMR	NRMR	RMR	NRMR	RMR	NRMR	RMR	NRMR	RMR
194	67	2	8	NA	NA	51	42	49	34	NA	NA
1085	133	NA	NA	384	62	NA	NA	NA	NA	NA	NA
742	98	9	29	179	36	NA	NA	NA	NA	50	6
195	25	4	1	NA	NA	63	14	59	13	37	17
659	133	1	40	34	16	NA	NA	NA	NA	NA	NA
35	36	NA	NA	NA	NA	NA	NA	NA	NA	9	22
46	10	2	1	1	2	NA	NA	NA	NA	NA	NA
240	34	0	0	3	0	NA	NA	NA	NA	NA	NA
62	15	1	3	18	10	28	9	NA	NA	NA	NA
58	20	0	2	1	2	NA	NA	5	4	NA	NA
79	25	NA	NA	3	1	NA	NA	NA	NA	36	19
16	10	2	2	NA	NA	3	4	1	2	NA	NA
37	16	1	1	NA	NA	NA	NA	NA	NA	NA	NA
19	41	0	21	NA	NA	NA	NA	NA	NA	NA	NA
63	12	0	1	13	1	NA	NA	NA	NA	NA	NA
104	35	0	13	NA	NA	NA	NA	NA	NA	NA	NA
95	14	3	2	5	5	NA	NA	NA	NA	NA	NA
125	44	1	16	NA	NA	NA	NA	NA	NA	NA	NA
612	89	7	20	NA	NA	NA	NA	NA	NA	NA	NA
109	42	6	6	15	7	NA	NA	NA	NA	NA	NA
51	19	NA	NA	NA	NA	NA	NA	NA	NA	3	2
218	42	4	1	8	1	NA	NA	NA	NA	NA	NA

MR: mitral regurgitation; MVr: mitral valve repair; NA: not available; NRMR: no recurrent mitral regurgitation; NYHA III/IV: New York Heart Association functional class III/IV; RMR: recurrent mitral regurgitation; TMVr: transcatheter mitral valve repair.

### Reoperations

**Figure 2:**
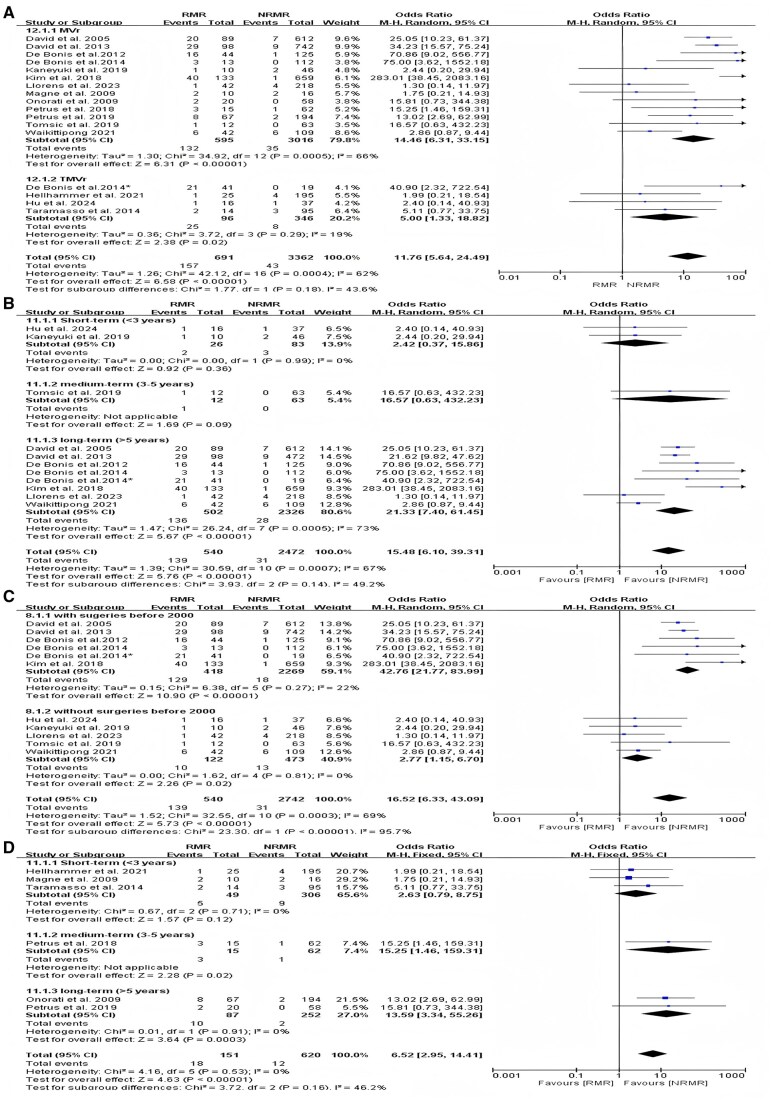
(**A**) The reoperation rates for patients with recurrent mitral regurgitation compared to those without recurrent mitral regurgitation in a subgroup analysis based on the surgical approach (MVr and TMVr). (**B**) The subgroup analysis based on follow-up time in studies about primary mitral regurgitation. (**C**) The subgroup analysis based on the year of the operation in studies about primary mitral regurgitation. (**D**) The subgroup analysis based on follow-up time in studies about secondary mitral regurgitation. CI: confidence interval; MVr: mitral valve repair; NRMR: no recurrent mitral regurgitation; RMR: recurrent mitral regurgitation; TMVr: transcatheter mitral valve repair.

Seventeen studies reported the occurrence of reoperations during follow-up in patients with recurrent MR compared to those without recurrent MR after intervention. The subgroup analysis based on surgical approach (MVr and TMVr) showed that regardless of whether patients underwent MVr [odds ratio (OR) = 14.46, 95% CI, 6.31–33.15; *P* < 0.001, *I*^2^ = 66%] or TMVr (OR = 5.0, 95% CI, 1.33–18.82; *P* = 0.029, *I*^2^ = 19%), postoperative recurrent MR was significantly associated with increased postoperative cardiovascular death. The overall OR was 11.76 (95% CI, 5.64–24.49; *P* < 0.001), with a high heterogeneity of 62% (Fig. 2A).

Patients in 11 studies had primary MR. For patients with primary MR, subgroup analysis was performed based on follow-up duration: short-term (<3 years), mid-term (3–5 years) and long-term (>5 years). The results showed that recurrent MR influences the incidence of reoperation during the follow-up period, and patients with recurrent MR are more likely to undergo reoperation than those without (OR = 15.48, 95% CI, 6.10–39.31; *P* < 0.001) (Fig. 2B). The heterogeneity in short-term and long-term subgroups is 0% and 73%, respectively. Besides, the groups of patients with primary MR had high heterogeneity overall (*I*^2 ^= 67%). Considering the high heterogeneity in every subgroup, we chose alternative grouping criteria. We found that some studies included patients who underwent intervention before 2000, whereas others included patients who had operations in 2003 or later. So, we divided the patients into 2 subgroups: those who had operations before 2000 and those without. The OR for the former subgroup was 50.78 (95% CI, 29.54–87.28; *P* < 0.001), and the OR for the latter subgroup was 2.78 (95% CI, 1.19–6.50; *P* = 0.02 < 0.05) (Fig. 2C). The heterogeneity between the groups was low for the 2 subgroups: *I*^2^ = 22% and *I*^2^ = 0%. Meta-regression analysis of the subgroups showed *P* = 0.034 < 0.05, indicating that some studies included patients who underwent intervention before 2000, which is a significant influencing factor for heterogeneity, consistent with the subgroup analysis results. The results of the subgroup analysis further confirmed that the reoperation rate was higher in patients with recurrent MR. An examination of funnel plots revealed symmetry (Supplementary Fig. S1). The application of Egger’s test revealed no publication bias (*P* = 0.664 > 0.05).

In 6 of the 18 articles, the issue of reoperation for recurrent MR after intervention in patients with secondary MR was discussed. In the subgroup analysis based on follow-up time, patients with secondary MR also had the same result with a significant correlation between recurrent MR and re-operative treatment (OR = 6.52, 95% CI, 2.95–14.41; *P* < 0.001) (Fig. 2D), and there was no heterogeneity between the studies (*I*^2^ = 0%). An examination of funnel plots revealed symmetry (Supplementary Fig. S2). The application of Egger’s test showed no publication bias (*P* = 0.852 > 0.05).

### Cardiovascular deaths

**Figure 3:**
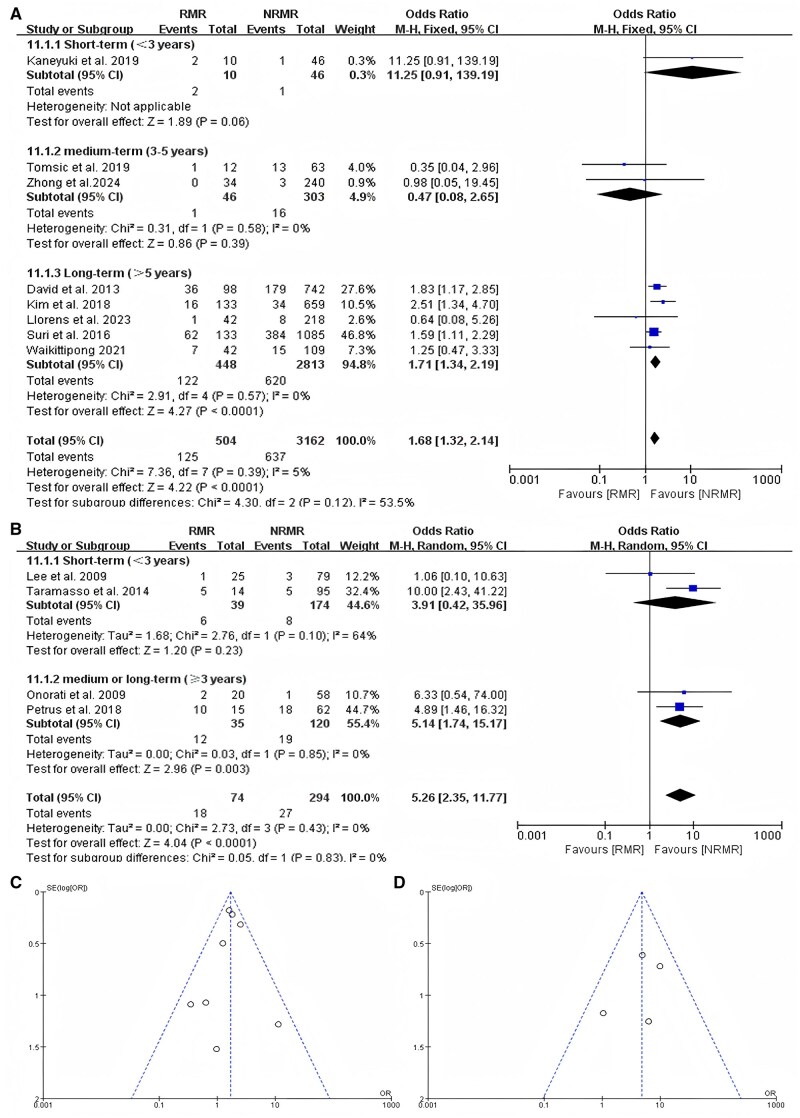
(**A**) The mortality of patients with recurrent mitral regurgitation compared to those without recurrent mitral regurgitation in a subgroup analysis based on follow-up time in studies about primary mitral regurgitation. (**B**) The mortality of patients with recurrent MR compared to those without recurrent MR in a subgroup analysis based on follow-up time in studies about secondary mitral regurgitation. (**C**) Funnel plot analysis of primary mitral regurgitation group. (**D**) Funnel plot analysis of secondary mitral regurgitation group. CI: confidence interval; M-H: Mantel-Haenszel method; NRMR: no recurrent mitral regurgitation; RMR: recurrent mitral regurgitation.

A total of 4035 patients in 12 studies reported the impact of recurrent MR on the cardiovascular deaths of patients after MVr, with the mean follow-up period ranging from 1.06 to 11.5 years. In 8 studies of patients with primary MR, the subgroup analysis based on short-term (<3 years), medium-term (3–5 years) and long-term (>5 years) follow-up showed that recurrent MR was significantly associated with increased postoperative cardiovascular deaths (OR = 1.68, 95% CI, 1.32–2.14; *P* < 0.001) (Fig. 3A). Heterogeneity in all subgroups was 0%, whereas overall heterogeneity was 5%. The examinations of funnel plots revealed symmetry (Fig. 3C). The results of the studies included patients with primary regurgitation (*P* = 0.647 > 0.05) who did not exhibit publication bias, according to the application of Egger’s test.

Four studies of patients with secondary MR showed the same outcome as those with primary MR in 8 studies. The cardiovascular death rate was higher in patients with recurrent MR compared to those with non-recurrent MR in the subgroup analysis (OR = 5.26, 95% CI, 2.35–11.77; *P* < 0.001) (Fig. 3B), and the overall heterogeneity was 0%. The examinations of funnel plots also revealed symmetry (Fig. 3D), and patients with secondary MR (*P* = 0.533 > 0.05) did not exhibit publication bias in Egger’s test.

### Readmission

**Figure 4:**
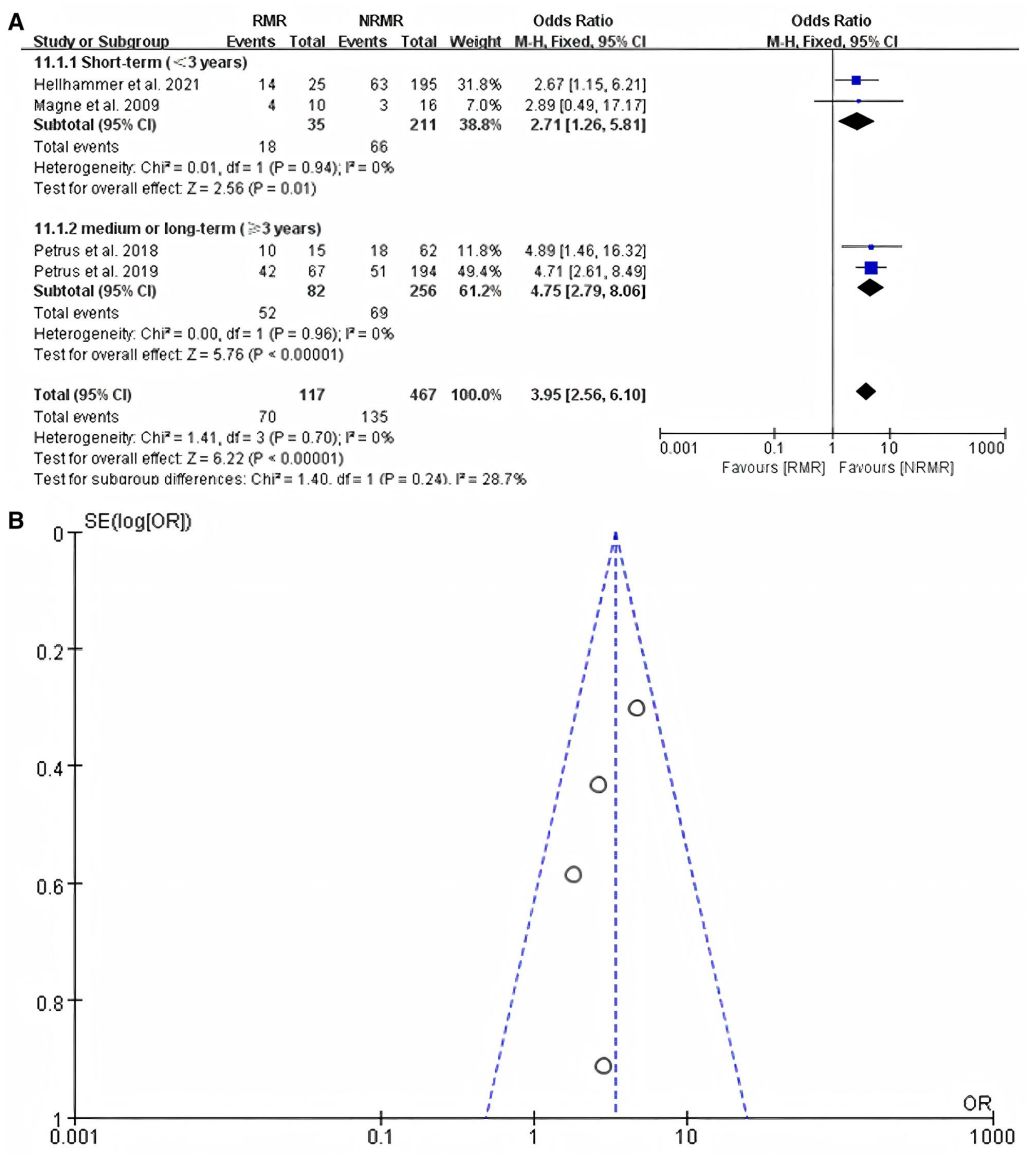
(**A**) The readmission rates for patients with recurrent mitral regurgitation compared to those without recurrent mitral regurgitation in a subgroup analysis based on follow-up time in studies about secondary mitral regurgitation. (**B**) Funnel plot analysis. CI: confidence interval; M-H: Mantel-Haenszel method; NRMR: no recurrent mitral regurgitation; RMR: recurrent mitral regurgitation.

About 4 studies involving 584 patients with secondary MR reported the readmission rate of patients with recurrent MR and patients with non-recurrent MR. The subgroup analysis based on short-term (<3 years) and mid-to-long-term (≥3 years) follow-up showed results of OR = 2.71 (95% CI, 1.26–5.81; *P* = 0.01) and OR = 4.75 (95% CI, 2.79–8.06; *P* < 0.001), respectively. The overall OR was 3.95 (95% CI, 2.56–6.10; *P* < 0.001), with no heterogeneity (*I*^2^ = 0) in all subgroups (Fig. 4A). An examination of funnel plots revealed symmetry (Fig. 4B). The application of Egger’s test revealed no publication bias (*P* = 0.29 > 0.05).

### Heart failure

**Figure 5:**
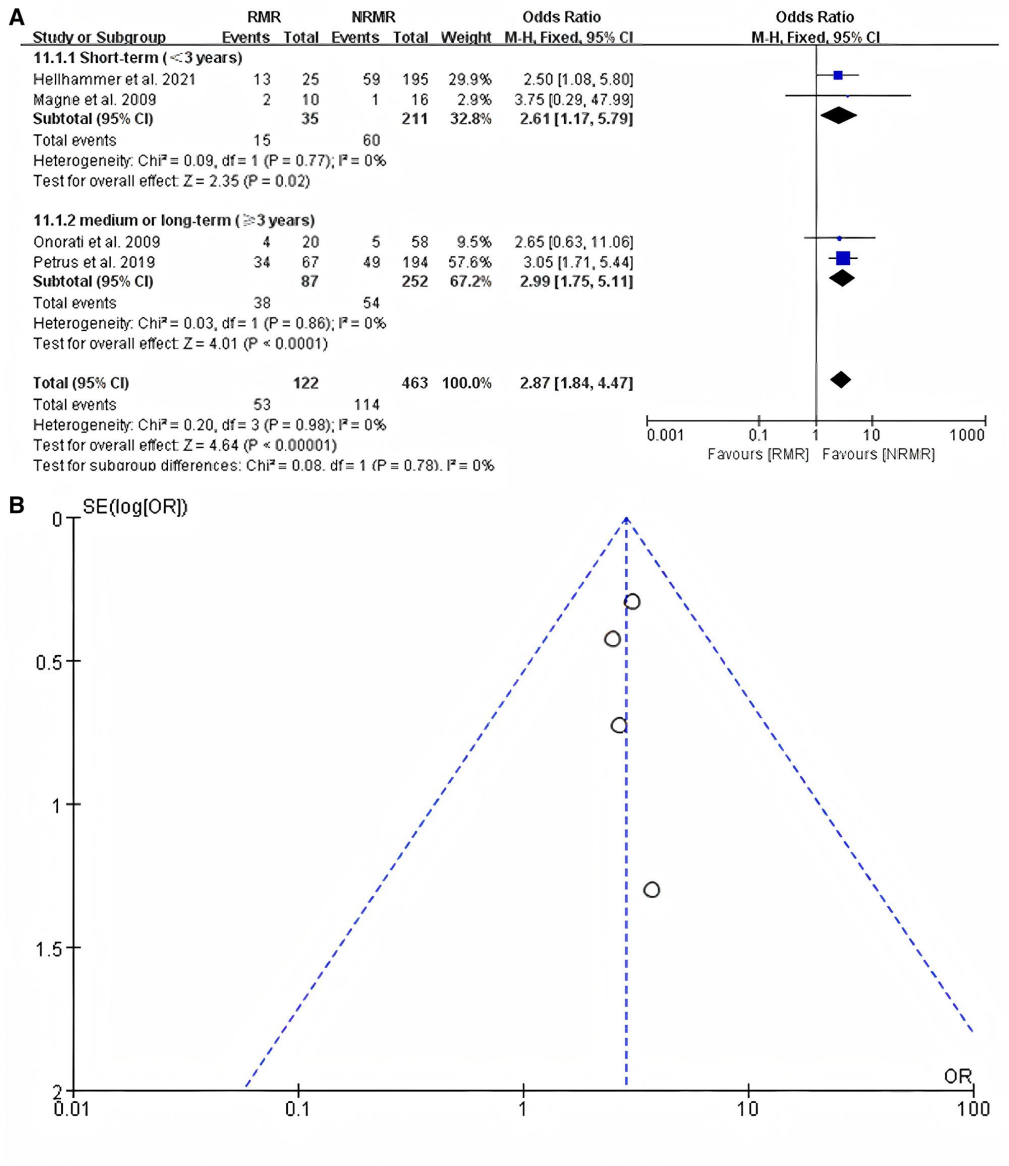
(**A**) The heart failure rates for patients with recurrent mitral regurgitation compared to those without recurrent mitral regurgitation in a subgroup analysis based on follow-up time in studies about secondary mitral regurgitation; (**B**) funnel plot analysis. NRMR: no recurrent mitral regurgitation; RMR: recurrent mitral regurgitation.

A total of 4 studies reported the incidence of heart failure, and the patients involved in the 4 studies were all those with secondary MR. For patients with secondary MR, those who experience recurrent MR after MVr are more likely to develop heart failure, whether in the short-term (<3 years) subgroup or in the mid-to-long-term (≥3 years) subgroup. The overall OR was 2.87 (95% CI, 1.75–5.11; *P* < 0.001) (Fig. 5A). Furthermore, the studies had no heterogeneity (*I*^2^ = 0%). An examination of funnel plots revealed symmetry (Fig. 5B). The application of Egger’s test revealed no publication bias (*P* = 0.995 > 0.05).

### New York Heart Association functional class III/IV

**Figure 6:**
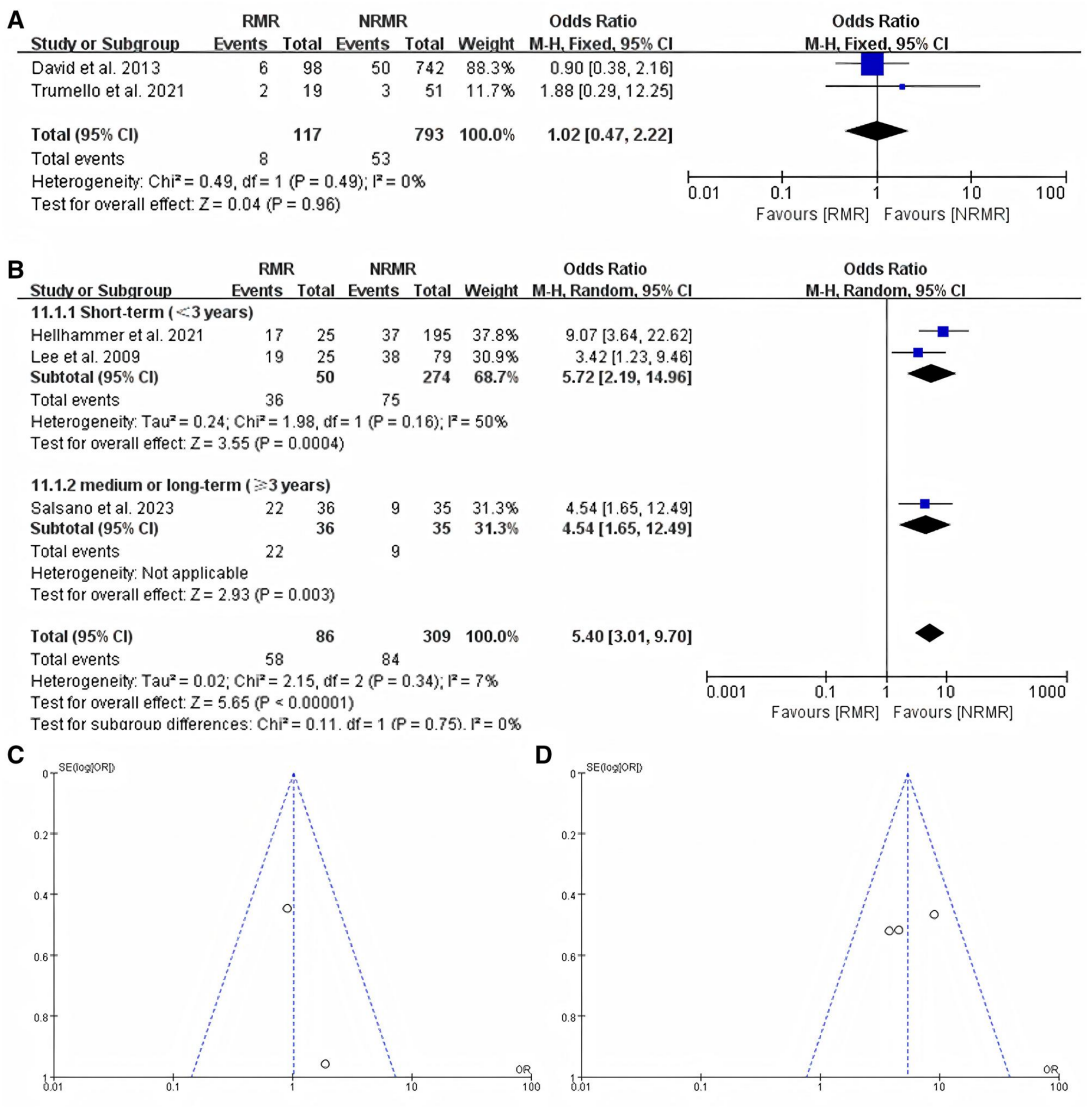
(**A**) New York Heart Association functional class III/IV of patients with recurrent mitral regurgitation compared to those without recurrent mitral regurgitation in studies about primary mitral regurgitation. (**B**) New York Heart Association functional class III/IV designation of patients with recurrent mitral regurgitation compared to those without recurrent mitral regurgitation in a subgroup analysis based on follow-up time in studies about secondary mitral regurgitation. (**C**) Funnel plot analysis of the primary mitral regurgitation group. (**D**) Funnel plot analysis of the secondary mitral regurgitation group. CI: confidence interval; NRMR: no recurrent mitral regurgitation; RMR: recurrent mitral regurgitation.

Five studies reported the incidence of NYHA functional class III/IV in patients with recurrent MR after intervention, 2 of which involved patients with primary MR. The results of 2 studies indicated no significant correlation between postoperative recurrent MR and the development of NYHA functional class III/IV (OR = 1.02, 95% CI, 0.47–2.22; *P* = 0.96) (Fig. 6A). *I*^2^ = 0 indicated that there was no potential heterogeneity across the studies. The examinations of funnel plots revealed symmetry (Fig. 6C).

Three studies involving secondary MR reported the incidence of NYHA functional class III/IV in patients with recurrent MR after intervention. In the subgroup analysis based on follow-up time, the results of the studies about secondary MR indicated that recurrent MR is associated with an increased risk of developing NYHA functional class III/IV in postoperative patients and can increase the incidence of NYHA functional class III/IV (OR = 5.40, 95% CI, 3.01–9.70; *P* < 0.001) (Fig. 6B), with low heterogeneity among the studies (*I*^2^ = 7%). The examinations of funnel plots revealed symmetry (Fig. 6D). The application of Egger’s test revealed no publication bias (*P* = 0.082 > 0.05).

## DISCUSSION

Recurrent MR has been shown to negatively impact clinical outcomes, including a decrease in postoperative quality of life and overall survival rates [9, 18]. However, its other effects on patients recovering from MVr or TMVr intervention have received little attention; most publications have concentrated on predicting recurrent MR and how it affects overall survival [26, 31, 45, 46].

This meta-analysis builds on previous studies to summarize the effect of recurrent MR on clinical outcomes after mitral valve interventions. Five factors are compared between patients with MR who had a recurrence and those who did not: the reoperation rates, cardiovascular deaths, readmission, heart failure and NYHA functional class III/IV. In our study, results show that in research involving secondary MR, patients with recurrent MR are more likely to undergo reoperation, experience cardiovascular death, be readmitted, have heart failure and be classified as NYHA functional class III/IV. The data from primary MR studies also show results similar to those of secondary MR, except for the NYHA functional class III/IV classification. The main outcome data for primary and secondary MR are presented in Supplementary Table S2. Additionally, whether patients received TMVr or MVr treatment did not affect these results. The meta-regression analysis was performed to examine the relationship between follow-up duration and the incidence of recurrent MR, and the results showed that follow-up duration had no significant effect on the incidence of recurrent MR (*P* = 0.933). However, the subgroup analysis based on follow-up duration indicated that recurrent MR was associated with a significantly higher incidence of reoperation, cardiovascular death, readmission, heart failure and NYHA functional class III/IV events in mid-term and long-term follow-up compared to short-term follow-up. Therefore, it is recommended that follow-up and intervention for patients with recurrent MR be strengthened, especially in cases with longer follow-up durations.

Recent research [44, 47–49] concluded that there could be 2 causes of recurrence: the surgical technique used during the operation and the ongoing progression of valve disease. Petrus *et al.* [15] also mentioned in their study that the standardized surgical approach they used, which involved implanting a semi-rigid annuloplasty ring downsized by 2 ring sizes and aiming for a coaptation length of at least 8 mm, resulted in fewer patients experiencing recurrent MR. Regardless of whether patients had primary or secondary MR, we found in this meta-analysis that patients with recurrent MR after operations had a much higher reoperation rate than patients without recurrent MR. This finding is consistent with data from other studies [15, 29, 33, 38, 44, 47].

There are many reasons for reoperations in these patients, such as ring dehiscence, endocarditis, progressive mitral leaflet tethering and younger age of surgical patients, with a significant portion attributed to recurrent MR, especially severe recurrent MR [15, 27, 29, 33, 47]. Additionally, some patients with moderate recurrent MR complicated by other cardiac diseases also require repeat operative intervention [36]. Compared to patients without recurrent MR, those with recurrent MR have a relatively higher cardiovascular death rate compared with both primary and secondary MR patients, with the majority of deaths attributed to cardiac-related causes, as shown in other studies [18, 30, 35, 47]. Furthermore, recurrent MR is associated with low survival rates and a 3 times higher risk of death [15, 26, 28, 48]. However, the study of Zhong *et al.* [32] showed that the mortality rates for both the recurrence group and the non-recurrence group are similar, which may be attributed to the low incidence of recurrent MR and mortality caused by surgical procedures.

Secondary MR patients with recurrent MR often experience the absence of ventricular reverse remodelling or even adverse reverse remodeling [50]. In the studies by Hung *et al.* [24] and Magne *et al.* [28], there is a significant increase in the end-diastolic volume, end-systolic volume and end-systolic sphericity index of the ventricle in patients with MR. Additionally, echocardiographic results indicate that postoperative improvement is poor in these patients [30, 51]. Therefore, compared to patients without recurrent RM, those with preoperative heart failure and NYHA functional class III/IV showed no significant improvement after an operation in the recurrent MR group [15, 21, 26, 30, 31]. This result also suggests that the proportion of heart failure and the number of patients with NYHA functional class III/IV is higher among those with recurrent MR, which is consistent with the findings of this meta-analysis. It has been found that patients with recurrent MR have a higher rate of requiring reoperation and experiencing heart failure, indicating that they require multiple readmissions for related treatments [15, 21, 27, 28].

In the studies by Petrus *et al.* [15], patients with recurrent MR were readmitted for treatment approximately 16.8 times per 100 patient-years, significantly higher than the 7.2 times per 100 patient-years for patients without recurrent MR. The results of our study further confirm this outcome. For patients with primary MR, our study results indicate that MR is not significantly correlated with the incidence of NYHA functional class III/IV, which differs from findings in other studies [40, 48], which may be attributed to the limited number of relevant studies included (only 2) and subjective non-uniform assessment of the NYHA functional class. In the 2 included studies, 1 study had a follow-up period of 1 year, whereas the other study extended beyond 10 years—such substantial variability in follow-up duration may further compromise the comparability of outcomes.

### Limitations

It is important to recognize a few limitations of the findings of this meta-analysis. First, the studies included in our research were retrospective observational studies, which inherently carry design biases. Secondly, except for a few studies, most included a relatively small number of patients, and many provided insufficient data; only a few reported heart failure rates and readmission rates in patients with recurrent MR, and most studies did not provide detailed information on disease-free survival rates or echocardiographic changes. Third, all of the heart failure and readmission rates statistics included studies focused on patients with secondary MR because data from patients with primary MR were lacking. Additionally, in the analysis of the incidence of NYHA functional class III/IV, only 2 studies involved patients with primary MR, which limits the reliability of the results. More research data will be needed in the future to confirm these findings. Moreover, only a limited number of studies included in this review provided specific patient counts for each grade of recurrent regurgitation, and studies focusing on post-TMVr outcomes are lacking. Therefore, we look forward to more data and research in the future to enable more detailed analyses of each grade and post-TMVr outcomes. Fifth, our study did not adjust for confounding factors such as preoperative left ventricular function, baseline MR severity, annuloplasty ring selection, leaflet repair strategy and surgeon experience. Finally, we did not explore the structural predictors of recurrent MR or the preventive strategies to reduce recurrent MR, which still require further clarification through multiple large randomized controlled trials.

## CONCLUSION

This meta-analysis confirms that patients with primary or secondary MR with recurrent MR after intervention (surgery or transcatheter) have a higher risk of reoperation and death. In patients with secondary MR, postoperative recurrent MR is significantly correlated with high rates of readmission, heart failure and the incidence of NYHA functional class III/IV. However, in patients with primary MR, recurrent MR does not mean a higher incidence of NYHA functional class III/IV. Recurrent MR is indeed a common complication following MVr or TMVr and can significantly impact postoperative patients in various aspects.

## Supplementary Material

ivaf109_Supplementary_Data

## Data Availability

The data underlying this article will be shared on reasonable request to the corresponding author.

## ABBREVIATIONS

CI: Confidence interval
MR: Mitral regurgitation
MVr: Mitral valve repair
NYHA: New York Heart Association
OR: Odds ratio
TMVr: Transcatheter mitral valve repair
