# Supplementary material for: The impact of recurrent mitral regurgitation after surgical or transcatheter mitral valve repair: a comprehensive review and a meta-analysis
Source: Interdiscip Cardiovasc Thorac Surg. 2025 Apr 29;40(6):ivaf109. doi: 10.1093/icvts/ivaf109 (PMC12141202; doi:10.1093/icvts/ivaf109)
Supplement: ivaf109_Supplementary_Data [file ivaf109_supplementary_data.zip › Supplementary materials.docx]

**Supplementary materials**

**The strategy of searching PubMed**

The database was searched on September 20, 2024, n=992.

Search Strategy:

1. “recurrent mitral regurgitation”[Title/Abstract]
2. “Mitral Valve repair”[Mesh]
3. “Mitral Valve Annulus Repair”[Title/Abstract]
4. “Mitral Annuloplasties”[Title/Abstract]
5. “Mitral Valve Annuloplasties”[Title/Abstract]
6. “Valve Annuloplasties, Mitral”[Title/Abstract]
7. “Valve Annuloplasty, Mitral”[Title/Abstract]
8. “Mitral Annuloplasty”[Title/Abstract]
9. 2 OR 3-8
10. ‘transcatheter mitral valve repair”[Title/Abstract]
11. 1 AND 9
12. 11 OR 10

(recurrent mitral regurgitation[Title/Abstract]) AND ((mitral valve repair[Mesh] OR Mitral Valve Annulus Repair[Title/Abstract] OR Mitral Annuloplasties[Title/Abstract] OR Mitral Valve Annuloplasties[Title/Abstract] OR Valve Annuloplasties, Mitral[Title/Abstract OR Valve Annuloplasty, Mitral[Title/Abstract] OR Mitral Annuloplasty[Title/Abstract]) OR( transcatheter mitral valve repair[Title/Abstract]))

**Web of Science**

The database was searched on September 20, 2024, n=703.

Search Strategy:

((TS=(recurrent mitral regurgitation)) AND TS=(Mitral Valve repair OR annuloplastic, Mitral Valve OR Annuloplasty, Mitral Valve OR Mitral Valve annuloplastic OR Valve annuloplastic, Mitral OR Valve Annuloplasty, Mitral OR Mitral Annuloplasty OR annuloplastic, Mitral OR Annuloplasty, Mitral OR Mitral annuloplastic OR Mitral Valve Annulus Repair))

**EMBASE**

The database was searched on September 20, 2024, n=261.

Search Strategy:

'recurrent mitral regurgitation':ti,ab,kw AND ('mitral valve repair':ti,ab,kw OR 'annuloplastic, mitral valve':ti,ab,kw OR 'annuloplasty, mitral valve':ti,ab,kw OR 'mitral valve annuloplastic':ti,ab,kw OR 'transcatheter mitral valve repair':ti,ab,kw OR 'mitral annuloplasty':ti,ab,kw OR 'annuloplasty, mitral':ti,ab,kw)

**Ovid MEDLINE**

The database was searched on September 20, 2024, n=695.

Search Strategy:

1.recurrent mitral regurgitation.ab.

2.mitral valve repair.ab.

3.transcatheter mitral valve repair.ab.

4.annuloplastic, Mitral Valve.ab.

5.Annuloplasty, Mitral Valve.ab.

6.Mitral Valve annuloplastic.ab.

7.Valve annuloplastic, Mitral.ab.

8.Valve Annuloplasty, Mitral.ab.

9.Mitral Annuloplasty.ab.

10.Annuloplasty, Mitral.ab.

11.Mitral annuloplastic.ab.

12.Mitral Valve Annulus Repair.ab.

13.or/2-12 [Segmentectomy]

14.1 and 13
