# Supplementary material for: The impact of recurrent mitral regurgitation after surgical or transcatheter mitral valve repair: a comprehensive review and a meta-analysis
Source: Interdiscip Cardiovasc Thorac Surg. 2025 Apr 29;40(6):ivaf109. doi: 10.1093/icvts/ivaf109 (PMC12141202; doi:10.1093/icvts/ivaf109)
Supplement: ivaf109_Supplementary_Data [file ivaf109_supplementary_data.zip › Supplementary Tables S1 to S2.docx]

**Supplementary Table S1: Definitions of Recurrent MR and Outcome Standards in Each Study**

| **Study Name** | **Definition of Recurrent MR** | **Reoperation Standard** | **Cardiovascular Death Standard** | **Rehospitalization Standard** | **Heart Failure Standard** | **NYHA Class III/IV Standard** |
| --- | --- | --- | --- | --- | --- | --- |
| **Petrus et al. (2019)** | **MR ≥ 2+ (moderate)** | **Surgery due to MR recurrence** | **Death due to cardiovascular causes** | **Readmission due to cardiovascular causes** | **Clinically confirmed heart failure** | **NYHA Class III/IV** |
| **Suri et al. (2016)** | **MR ≥ 2+ (moderate)** | **Surgery due to MR recurrence** | **Death due to cardiovascular causes** | **Readmission due to cardiovascular causes** | **Clinically confirmed heart failure** | **NYHA Class III/IV** |
| **David et al. (2013)** | **MR ≥ 2+ (moderate)** | **Surgery due to MR recurrence** | **Death due to cardiovascular causes** | **Readmission due to cardiovascular causes** | **Clinically confirmed heart failure** | **NYHA Class III/IV** |
| **Hellhammer et al. (2021)** | **MR ≥ 2+ (moderate)** | **Surgery due to MR recurrence** | **Death due to cardiovascular causes** | **Readmission due to cardiovascular causes** | **Clinically confirmed heart failure** | **NYHA Class III/IV** |
| **Kim et al. (2018)** | **MR ≥ 2+ (moderate)** | **Surgery due to MR recurrence** | **Death due to cardiovascular causes** | **Readmission due to cardiovascular causes** | **Clinically confirmed heart failure** | **NYHA Class III/IV** |
| **Salsano et al. (2023)** | **MR ≥ 2+ (moderate)** | **Surgery due to MR recurrence** | **Death due to cardiovascular causes** | **Readmission due to cardiovascular causes** | **Clinically confirmed heart failure** | **NYHA Class III/IV** |
| **Kaneyuki et al. (2019)** | **MR ≥ 2+ (moderate)** | **Surgery due to MR recurrence** | **Death due to cardiovascular causes** | **Readmission due to cardiovascular causes** | **Clinically confirmed heart failure** | **NYHA Class III/IV** |
| **Zhong et al. (2024)** | **MR ≥ 3+ (severe)** | **Surgery due to MR recurrence** | **Death due to cardiovascular causes** | **Readmission due to cardiovascular causes** | **Clinically confirmed heart failure** | **NYHA Class III/IV** |
| **Petrus et al. (2018)** | **MR ≥ 2+ (moderate)** | **Surgery due to MR recurrence** | **Death due to cardiovascular causes** | **Readmission due to cardiovascular causes** | **Clinically confirmed heart failure** | **NYHA Class III/IV** |
| **Onorati et al. (2009)** | **MR ≥ 2+ (moderate)** | **Surgery due to MR recurrence** | **Death due to cardiovascular causes** | **Readmission due to cardiovascular causes** | **Clinically confirmed heart failure** | **NYHA Class III/IV** |
| **Lee et al. (2009)** | **MR ≥ 2+ (moderate)** | **Surgery due to MR recurrence** | **Death due to cardiovascular causes** | **Readmission due to cardiovascular causes** | **Clinically confirmed heart failure** | **NYHA Class III/IV** |
| **Magne et al. (2009)** | **MR ≥ 2+ (moderate)** | **Surgery due to MR recurrence** | **Death due to cardiovascular causes** | **Readmission due to cardiovascular causes** | **Clinically confirmed heart failure** | **NYHA Class III/IV** |
| **Hu et al. (2024)** | **MR ≥ 2+ (moderate)** | **Surgery due to MR recurrence** | **Death due to cardiovascular causes** | **Readmission due to cardiovascular causes** | **Clinically confirmed heart failure** | **NYHA Class III/IV** |
| **De Bonis et al. (2014)** | **MR ≥ 3+ (severe)** | **Surgery due to MR recurrence** | **Death due to cardiovascular causes** | **Readmission due to cardiovascular causes** | **Clinically confirmed heart failure** | **NYHA Class III/IV** |
| **Tomsic et al. (2019)** | **MR ≥ 2+ (moderate)** | **Surgery due to MR recurrence** | **Death due to cardiovascular causes** | **Readmission due to cardiovascular causes** | **Clinically confirmed heart failure** | **NYHA Class III/IV** |
| **Taramasso et al. (2014)** | **MR ≥ 2+ (moderate)** | **Surgery due to MR recurrence** | **Death due to cardiovascular causes** | **Readmission due to cardiovascular causes** | **Clinically confirmed heart failure** | **NYHA Class III/IV** |
| **De Bonis et al. (2012)** | **MR ≥ 3+ (severe)** | **Surgery due to MR recurrence** | **Death due to cardiovascular causes** | **Readmission due to cardiovascular causes** | **Clinically confirmed heart failure** | **NYHA Class III/IV** |
| **De Bonis et al. (2014)** | **MR ≥ 3+ (severe)** | **Surgery due to MR recurrence** | **Death due to cardiovascular causes** | **Readmission due to cardiovascular causes** | **Clinically confirmed heart failure** | **NYHA Class III/IV** |
| **David et al. (2005)** | **MR ≥ 2+ (moderate)** | **Surgery due to MR recurrence** | **Death due to cardiovascular causes** | **Readmission due to cardiovascular causes** | **Clinically confirmed heart failure** | **NYHA Class III/IV** |
| **Waikittipong (2021)** | **MR ≥ 2+ (moderate)** | **Surgery due to MR recurrence** | **Death due to cardiovascular causes** | **Readmission due to cardiovascular causes** | **Clinically confirmed heart failure** | **NYHA Class III/IV** |
| **Trumello et al. (2021)** | **MR ≥ 2+ (moderate)** | **Surgery due to MR recurrence** | **Death due to cardiovascular causes** | **Readmission due to cardiovascular causes** | **Clinically confirmed heart failure** | **NYHA Class III/IV** |
| **Llorens et al. (2019)** | **MR ≥ 2+ (moderate)** | **Surgery due to MR recurrence** | **Death due to cardiovascular causes** | **Readmission due to cardiovascular causes** | **Clinically confirmed heart failure** | **NYHA Class III/IV** |

**Supplementary Table S2: Main Outcome Data for Primary and Secondary MR**

1. **† Indicates a statistically significant association in patients with secondary MR (P < 0.05).**
2. **Bolded OR values highlight a significantly increased risk (95% CI does not cross 1).**

| **Outcome** | **MR Type** | **Number of Studies** | **Total Patients** | **Number of Events** | **OR** | **95% CI** | **P-value** |
| --- | --- | --- | --- | --- | --- | --- | --- |
| Reoperation Rate | Primary**†** | 11 | 4,858 | 748 | 22.54 | **14.96–33.98** | <0.001 |
|  | Secondary**†** | 6 | 946 | 212 | 6.25 | **2.95–14.41** | <0.001 |
| Cardiovascular Mortality | Primary**†** | 8 | 4,858 | 748 | 1.68 | **1.32–2.14** | <0.001 |
|  | Secondary**†** | 4 | 946 | 212 | 5.26 | **2.35–11.77** | <0.001 |
| Rehospitalization Rate | Secondary**†** | 4 | 584 | 212 | 3.95 | **2.56–6.10** | <0.001 |
| Heart Failure Rate | Secondary**†** | 4 | 584 | 212 | 2.87 | **1.75–5.11** | <0.001 |
| NYHA Class III/IV | Primary | 2 | 4,858 | 748 | 1.02 | **0.47–2.22** | 0.96 |
|  | Secondary**†** | 3 | 946 | 212 | 5.40 | **3.01–9.70** | <0.001 |
