# Supplementary figures and images for: The impact of recurrent mitral regurgitation after surgical or transcatheter mitral valve repair: a comprehensive review and a meta-analysis
Source: Interdiscip Cardiovasc Thorac Surg. 2025 Apr 29;40(6):ivaf109. doi: 10.1093/icvts/ivaf109 (PMC12141202; doi:10.1093/icvts/ivaf109)

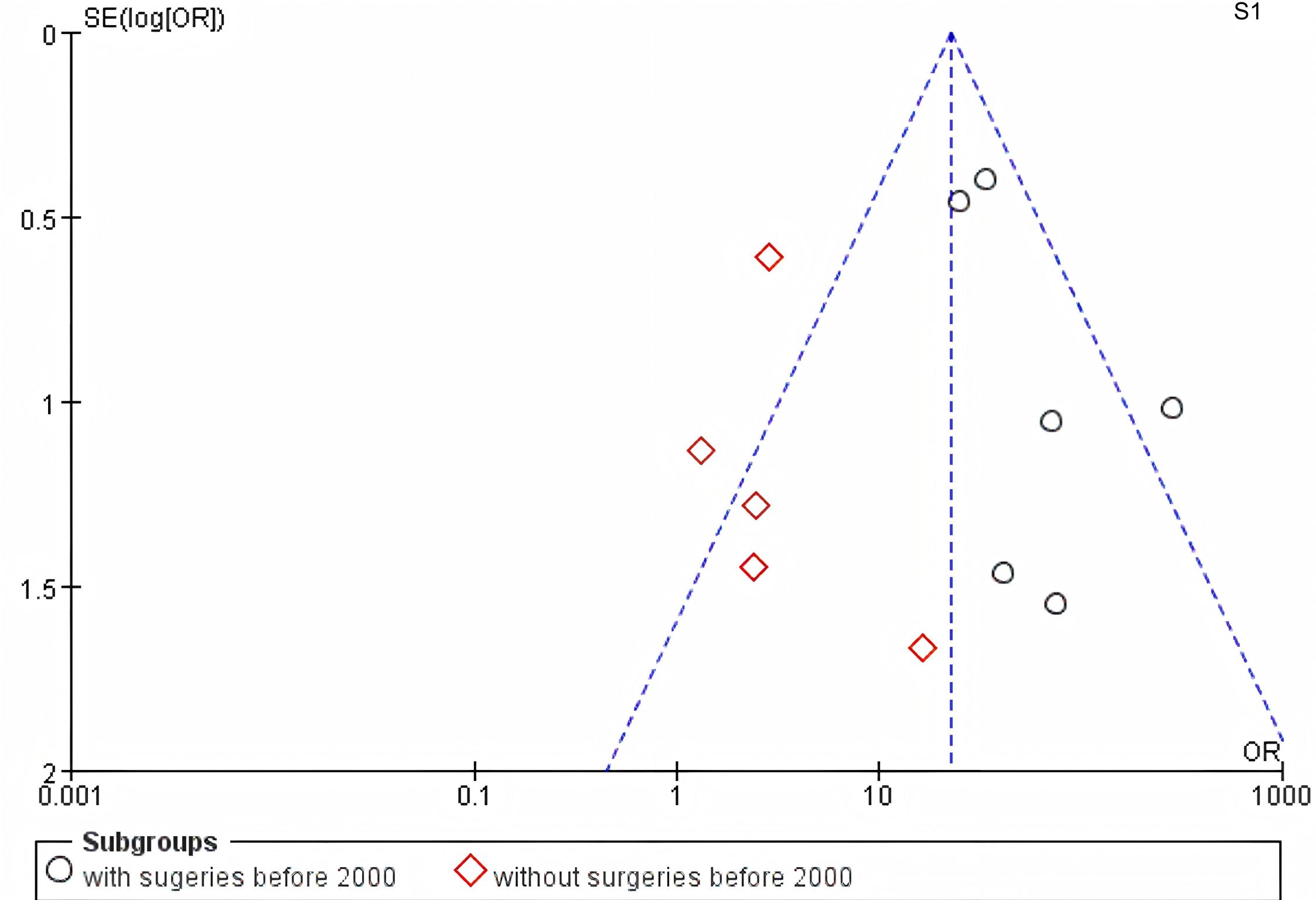

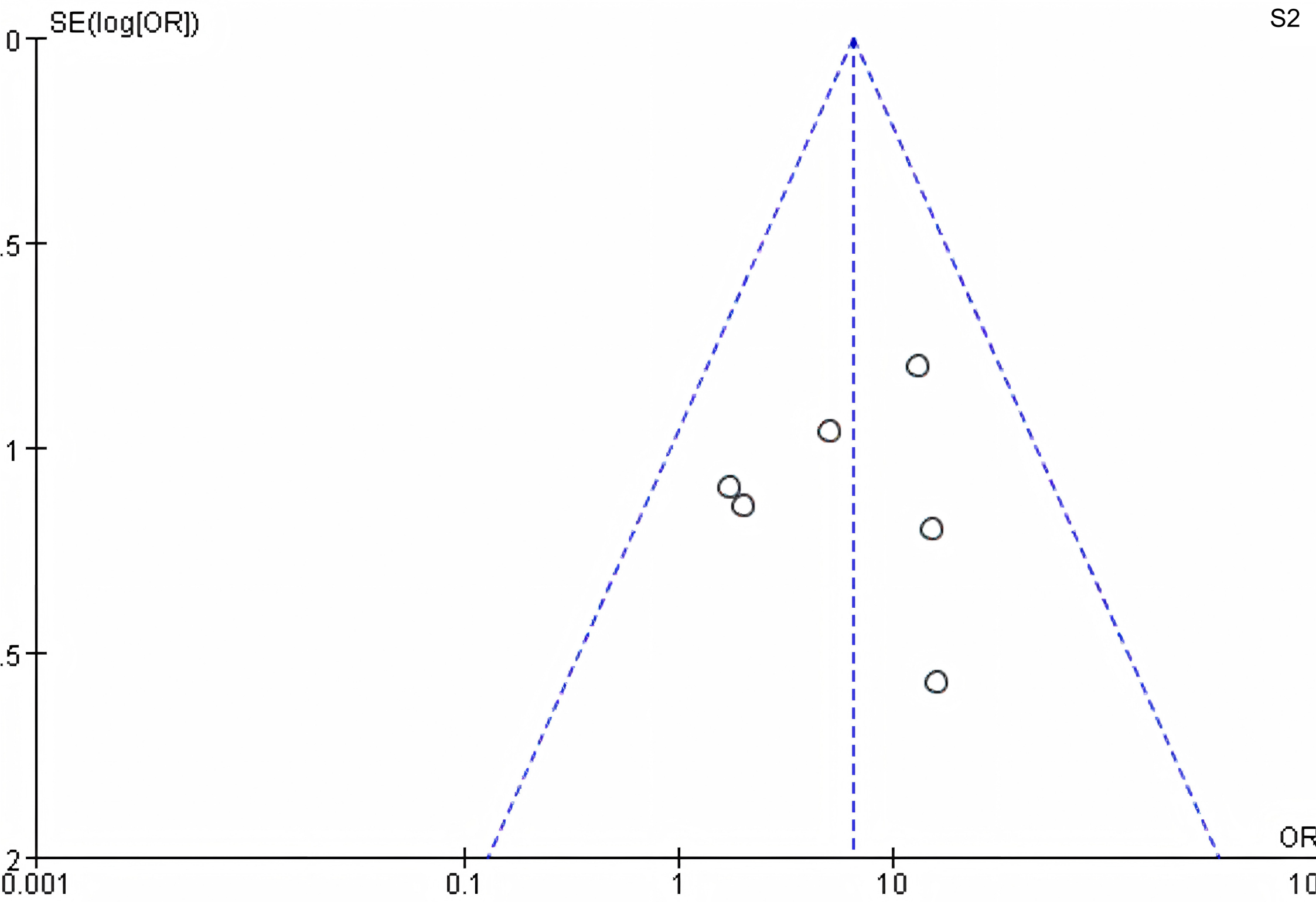

Supplement: ivaf109_Supplementary_Data [file ivaf109_supplementary_data.zip › Supplementary Figures S1 to S2.pdf]
